# Diabetes and ischemic heart disease: double jeopardy with regard to depressive mood and reduced quality of life

**DOI:** 10.1530/EC-14-0083

**Published:** 2014-09-16

**Authors:** Natasha Bergmann, Søren Ballegaard, Pernille Holmager, Per Bech, Åke Hjalmarson, Finn Gyntelberg, Jens Faber

**Affiliations:** 1 Department of Endocrinology, Herlev University Hospital, Herlev, Denmark; 2 Ull Care A/S, Hellerup, Denmark; 3 Psychiatric Research Unit, Psychiatric Center North Zealand, Hillerød, Denmark; 4 The Cardiovascular Institute, Sahlgrenska University Hospital, Goteborg, Sweden; 5 The National Research Center for the Working Environment, Copenhagen, Denmark; 6 Faculty of Health and Medical Sciences, Copenhagen University, Copenhagen, Denmark

**Keywords:** diabetes, ischemic heart disease, psychological stress, depressive symptoms, well-being, pressure pain sensitivity

## Abstract

The aim of this study was to test i) whether patients having diabetes and ischemic heart disease (IHD), i.e., patients suffering from two chronic diseases, demonstrate a higher degree of chronic stress when compared with patients suffering from IHD alone, and ii) whether suffering from the two chronic diseases results in an elevation in specific elements of the chronic stress concept. A total of 361 participants with IHD were included, of whom 47 suffered from concomitant diabetes. Stress was measured by pressure pain sensitivity (PPS) and by the following questionnaires: the Major Depression Inventory (MDI), the SF-36 Quality of Life questionnaire (SF-36 QOL), the WHO-5 Well-being Index, and the clinical stress signs (CSSs) scale. Participants with diabetes and IHD had a higher MDI score, a lower SF-36 physical component summary score, and a lower score of several sub-measurements of the SF-36 mental component score when compared with patients with IHD without diabetes. No significant differences were observed regarding stress measured by the PPS measure, the WHO-5 Well-being Index, or the number of CSSs. In conclusion, the combination of diabetes and IHD seems to be associated with increased depressive symptoms, lower overall physical QOL, and reduced mental QOL on several sub-elements of the questionnaire. This should be recognized in the management of patients with double diagnoses.

## Introduction

The risk of depression in people with diabetes is two times higher when compared with patients without diabetes, and major depression and elevated depressive symptoms have been found to be present in 11 and 31%, respectively, of individuals suffering from diabetes (1). The 12-month odds ratio of major depression is also increased more than twice in outpatients after a myocardial infarction (MI) (2) and ∼20% of outpatients with stable coronary artery disease have depressive symptoms (Patient Health Questionnaire ≥10) (3). Similarly, psychosocial stress is increased both in patients with diabetes and in patients with ischemic heart disease (IHD), and stress is generally accepted to be a risk factor for a poor outcome in both patient groups (4, 5, 6). The combination of suffering from both diabetes and IHD is very common as the presence of diabetes doubles the age-adjusted risk for cardiovascular disease in men and triples it in women (7). However, the psychological impact of suffering from both chronic diseases simultaneously still needs to be evaluated.

A newly developed handheld device measuring stress-induced hyperalgesia as pressure pain sensitivity (PPS) has shown promising results as a new measure of chronic stress both alone and in combination with a score of clinical stress signs (CSSs) (8, 9). A high PPS has previously been found to correlate with well-known stress reactions such as blood pressure, pulse rate, and work of the heart in healthy individuals (9), and to correlate with elements of stress as depressive symptoms, reduced well-being, and reduced quality of life (QOL) in patients with IHD (8). Thus, PPS may be used as a semi-objective measure covering the whole concept of stress.

In this study, we questioned i) whether patients having both diabetes and IHD, i.e., patients suffering from two chronic diseases, demonstrate a higher degree of chronic stress when compared with patients suffering from IHD alone, as measured by PPS and validated questionnaires evaluating depressive symptoms, QOL, psychological well-being, and CSSs, and ii) whether suffering from the two chronic diseases results in an elevation in specific elements of the chronic stress concept when compared with patients suffering solely from IHD.

## Materials and methods

The materials and methods followed in this study have been described in detail previously (8). In short, this was a cross-sectional study of patients with established and stable IHD. The inclusion criteria were as follows: established IHD defined as having a previous MI, or percutaneous coronary intervention, or coronary artery bypass graft surgery) and having completed cardiac rehabilitation more than 6 months before inclusion. The exclusion criteria were as follows: age over 75 years; suffering from unstable IHD such as scheduled cardiac surgery or changes in heart medication within 1 month before inclusion; hospitalization due to psychiatric disease; and suffering from other chronic competing disorders, which clearly impaired the participant's QOL.

PPS was measured by an algometric device, which quantifies the induced sensitivity for pressure experienced when stressed. The measurement was performed by a professional instructor after ten minutes of rest in a supine position on the most sensitive point on the sternum in the area of IC 4–5 and has been described in detail previously (8, 9, 10).

The following questionnaires were used for evaluating the different elements of the chronic stress concept: the Major Depression Inventory (MDI), which measures depressive symptoms, with a higher score equaling more depressive symptoms; the SF-36 Quality of Life questionnaire (SF-36 QOL), measuring physical and psychological QOL with a higher score equaling a better QOL; the WHO-5 Well-being Scale (WHO-5), with a higher score measuring a higher psychological well-being; and a newly developed questionnaire, the Clinical Stress Scale questionnaire, CSS, which includes a score of 56 CSSs experienced during the last 4 weeks, with each stress sign equaling one point on the scale (8). The questionnaires were answered on a website established for the study (www.songheart.org), and each participant had a personal login, which was first opened after the study ended in order to avoid bias.

As this study aimed to evaluate the influence on stress caused by the knowledge of suffering from two chronic diseases, diabetes was diagnosed based on a self-reported history, i.e., the knowledge of having diabetes and not on biochemical cut-off levels.

Participants were recruited from a database on subjects with established IHD (HjerteRask) at the Departments of Cardiology at Gentofte and Herlev University Hospitals, Copenhagen, Denmark. Participants had been rehabilitated according to the national guidelines during the period of 1999–2011 and were recruited to this study between June 2011 and February 2012. Written informed consent was obtained from all participants and the study was approved by the local ethical committee and was registered on www.clinicaltrials.gov (NCT01513824).

## Results

A total of 361 participants with IHD were included, of whom 47 (13%) suffered from known and concomitant diabetes. Participants with both diabetes and IHD were comparable to participants with only IHD with respect to age, sex, and cardiovascular medication except for use of calcium antagonists (Table 1). Among patients with diabetes, 27 were treated with oral hypoglycemic agents (OHAs), and seven with insulin either alone or in combination with OHAs. Participants with diabetes and IHD had a higher MDI score (more depressive symptoms), a lower SF-36 physical component summary score, and a lower score of several sub-measurements of the SF-36 mental component summary score when compared with patients with IHD but without diabetes (Table 2). No significant differences were observed regarding stress measured by the WHO-5 Well-being index, by the number of CSSs or by the PPS measure (Table 2). No differences in stress measurements were found between the diabetes patients on OHAs only vs the insulin treated (*P*>0.05 for PPS, MDI, SF-36, WHO, and CSS).

## Discussion

This study demonstrates that the combination of diabetes and chronic and stable IHD seems to be associated with increased depressive symptoms, lower overall physical QOL, and reduced mental QOL on several sub-elements of the questionnaire when compared with patients with IHD not suffering from known diabetes.

The mean MDI in the Danish general population has been found to be 7.2 (11). Participants with IHD of this study had a mean MDI of 8.0 and participants with both diabetes and IHD an MDI of 11, suggesting a rather large increase in depressive symptoms in patients suffering from both chronic diseases. In the Danish general population, ∼11% has an MDI ≥15, suggestive of mild depression. In this study, this figure was 16% in patients with IHD and 23% in the group with both diabetes and IHD, supporting the increased burden of depressive symptoms found in this patient group.

In the present cohort, 58% had a PPS ≥60, which is regarded as a cut-off value for increased chronic stress (8). This figure was not different in relation to having both diabetes and IHD vs having only IHD. However, in an otherwise healthy population, only 27% have been found to have PPS ≥60, demonstrating that the present cohort in fact did show signs of elevated stress, i.e., PPS (9). PPS measures pain threshold, and reduced pain threshold (i.e., increased PPS) is observed in several chronic diseases with a secondary generalized pain sensation such as fibromyalgia, arthritis, and post-traumatic stress syndrome (12, 13). Among the sub-items of the physical SF-36 questionnaire was a question of body pain. This parameter was not changed due to the presence of diabetes, and it might be speculated if the reduced score of overall physical QOL is related not to pain in particular, but rather to reduced ability of motion in general, as general stiffness of connective tissue is a well-known entity of long-standing diabetes (14).

In a recent randomized study with 217 participants with IHD receiving either treatment as usual or a program with daily self-measurement of PPS followed by reflection and action if a high PPS was measured, we found that, after 3 months, the active group, measuring PPS, showed a significant decrease in both PPS and MDI, suggestive of lower stress and lower level of depressive symptoms, and an increase in WHO-5 as a measure of a higher psychological well-being score, when compared with the group receiving treatment as usual (10). Overall, the Cohen's effect size was 0.12 between the active and the control groups; however, the effect of the intervention was consistently more pronounced among the psychologically vulnerable subjects with a Cohen's effect size of 0.35 among the subjects with MDI ≥15 (an effect size similar to the effect of anti-depressive medication) and an effect size of 0.52 among subjects with both high PPS and CSSs (10).

A limitation to this study was the much smaller number of participants in the group with diabetes and IHD (*n*=47) when compared with the group with IHD alone (*n*=314), which should be acknowledged when interpreting the data. However, the groups were large enough to substantiate a trend toward a more vulnerable psychological profile among patients suffering from both diabetes and IHD. Distinction between patients with diabetes treated with only OHAs and with insulin was hampered by the small numbers, and the lack of differences between these groups should be taken with reservation.

Our findings should be acknowledged when dealing with patients with both diabetes and IHD, i.e., both from a diabetologist's and a cardiologist's perspectives. Based on our own study with stress intervention in patients with IHD mentioned above, stress-reducing therapies should be considered at least in patients with both diabetes and IHD. This finding might be broadened out to other clinical situations with combinations of chronic diseases, i.e., psychologically vulnerable patients, in which it is known that QOL is substantially reduced. This clearly calls for further studies.

In conclusion, this study finds that the combination of known diabetes and chronic and stable IHD seems to be associated with increased depressive symptoms, lower overall physical QOL, reduced mental QOL on several sub-elements, but not with reduced pain threshold as measured by PPS. We encourage clinicians to recognize the double jeopardy with regard to psychological and especially depressive symptoms when treating patients with double diagnoses of chronic nature.

## Figures and Tables

**Table 1:** Demographic data in patients with diabetes and ischemic heart disease (IHD) vs IHD alone.

	**IHD with diabetes** (*n*=47)	I**HD without diabetes** (*n*=314)	***P* value**
Men, *n* (%)	39 (83)	247 (79)	NS
Age, mean (s.d.)	64 (6.1)	63 (8.1)	NS
β-blockers, *n* (%)	32 (68)	184 (59)	NS
Diuretics, *n* (%)	19 (40)	89 (28)	NS
Cholesterol-lowering medication, *n* (%)	40 (85)	279 (89)	NS
Angiotensin II receptor blockers or ACE inhibitors, *n* (%)	32 (68)	175 (56)	NS
Calcium antagonists, *n* (%)	21 (45)	64 (20)	0.001

**Table 2:** Psychological status in patients with diabetes and ischemic heart disease (IHD) vs IHD alone. Data are expressed as mean (s.d.).

	**IHD with diabetes** (*n*=47)	**IHD without diabetes** (*n*=314)	***P* value**
**Major Depression Inventory** **a**	**11 (7.7)**	**8.0 (7.0)**	**0.019**
WHO-5 Well-being Index	62 (19)	67 (18)	0.070
SF-36 Quality of life score			
**Physical component summary score**	**45 (10)**	**49 (8.7)**	**0.001**
**Physical functioning **	**85 (16)**	**71 (24)**	**<0.001**
**Role** ** – ** **physical**	**65 (37)**	**80 (31)**	**0.003**
Bodily pain	78 (23)	81 (21)	0.326
Mental component summary score	52 (9.5)	53 (9.8)	0.442
**General health**	**58 (18)**	**68 (19)**	**0.001**
**Vitality**	**57 (25)**	**67 (20)**	**0.004**
Social functioning	90 (16)	92 (17)	0.443
**Role** ** – ** **emotional**	**69 (34)**	**83 (30)**	**0.004**
Mental health	78 (17)	80 (15)	0.264
Pressure pain sensitivity	63 (18)	65 (20)	0.558
Clinical stress signs score	10 (7.6)	8.1 (6.7)	0.073

a Bold text means *P*<0.005.
